# Enhancing soybean tolerance to pre-emergent herbicides via biochar seed coating for eco-safe food systems

**DOI:** 10.3389/fpls.2025.1700864

**Published:** 2026-01-19

**Authors:** Muhammad Awais Arshad, Rana Nadeem Abbas, Rania Baloch, Ali Ahmad, Usman Zulfiqar, Fasih Ullah Haider, Hossam S. El-Beltagi, Mashael Daghash Alqahtani, P. V. Vara Prasad

**Affiliations:** 1 Department of Agronomy, Faculty of Agriculture, University of Agriculture Faisalabad, Faisalabad, Pakistan; 2 Department of Botany, Faculty of Sciences, University of Agriculture Faisalabad, Faisalabad, Pakistan; 3 Arid Zone Research Institute (AZRI), Bhakkar, Pakistan; 4 Department of Agronomy, Faculty of Agriculture and Environment, The Islamia University of Bahawalpur, Bahawalpur, Pakistan; 5 State Key Laboratory of Black Soils Conservation and Utilization, Northeast Institute of Geography and Agroecology, Chinese Academy of Sciences, Changchun, China; 6 Agricultural Biotechnology Department, College of Agriculture and Food Sciences, King Faisal University, Al-Ahsa, Saudi Arabia; 7 Department of Biology, College of Science, Princess Nourah bint Abdulrahman University, Riyadh, Saudi Arabia; 8 Department of Agronomy, Kansas State University, Manhattan, KS, United States

**Keywords:** biochar-coated seeds, crop growth parameters, herbicide tolerance, quality parameters, susceptibility index, weed management

## Abstract

Soybean (*Glycine max* L.) is highly sensitive to herbicides, which limits the effectiveness of chemical weed control and poses challenges to sustainable production. Biochar, a porous, carbon-rich material with a strong capacity for herbicide adsorption, is commonly applied as a soil amendment; however, its potential use as a seed coating to protect soybean seedlings from herbicide injury remains largely unexplored. To address this gap, a two-year field study (2022-2023) was conducted at the Agronomic Farm, University of Agriculture Faisalabad, to evaluate the effectiveness of biochar-coated soybean seeds in enhancing herbicide tolerance and suppressing weeds. The experiment was laid out in a randomized complete block design with a factorial arrangement, comprising 12 treatments replicated three times. The treatments included biochar-coated seeds and normal seeds, combined with six weed control treatments: s-metolachlor + pendimethalin, s-metolachlor, fluizefop-p-butyl, haloxyfop-p-ethyl, weed-free, and a weedy check. Growth, yield, quality, and weed-related indices were recorded throughout the study. Results revealed that biochar-coated seeds combined with s-metolachlor + pendimethalin significantly reduced weeds dry weight (699.5 and 516.2 kg ha^-1^), lower susceptibility index (1.5 and 1.2) and higher seed yield (1879.21 and 1933.77 kg ha^-1^), protein content (34.7% and 35.3%) and oil content (19.3 and 19.8%) in 2022 and 2023, respectively, demonstrating the protective role of biochar against herbicide-induced stress. This treatment also resulted in a 3.12% to 3.95% higher weed control percentage, a 9.93% to 12.55% increase in weed persistence index, and a 3.25% higher weed control efficiency. Moreover, lower weed indices were recorded, with yield losses limited to only 6.66% and 8.93% compared to normal seeds. Overall, these findings confirm that biochar-coated seeds, combined with s-metolachlor and pendimethalin, effectively suppress weeds with minimal crop injury, while also improving protein, oil content, and yield. This highlight biochar-based seed coating as a promising, climate-smart, and environmentally safe strategy for sustainable soybean production, warranting further investigation across diverse agro-ecological conditions.

## 1 Introduction

Soybean (*Glycine max* L.) is a globally important leguminous crop valued for its high nutritional and economical significance. It contains approximately 18-22% oil and 40-42% protein, along with essential vitamins and carbohydrates (Zhao et al., 2021). In Pakistan, soybean cultivation remains limited, and the country is heavily dependent on imports of soybeans, oilseeds, and edible oils, which consume substantial foreign exchange reserves. The rapid growing demand for soy-based products in the food industry, poultry feed, livestock nutrition, and aquaculture highlights an urgent need to enhance domestic soybean production. However, several constraints hinder its successful cultivation, including the lack of high-yielding, and well-adapted genotypes, suboptimal production practices, environmental stresses, and the adverse impacts of climate change. Among these constraints, weed infestation is one of the most critical challenges, with the potential to reduce yields by up to 60% (Asad et al., 2020). Early-season weed competition is particularly damaging, with reported yield losses ranging from 31% to 84% due to competition for light, water, and nutrients (Kachroo et al., 2003). Therefore, effective weed management is essential for sustaining soybean productivity.

Weed control is generally achieved through manual weeding or the application of herbicides. Although, manual weeding is environmentally friendly, it is labor-intensive and costly (Webster et al., 1999). In contrast, pre-emergence herbicides are more efficient, as they prevent weed seed germination and reduce early crop-weed competition (Tursun et al., 2016; Butts et al., 2017). Furthermore, the sequential application of pre- and post-emergence herbicides with different modes of action enhances weed control efficacy and helps delay the development of herbicide resistance (Norsworthy et al., 2012; Kumar and Jha, 2015; Oliveira et al., 2017).

Soybean plants are highly sensitive to herbicide applications, making the search for alternative protective strategies essential. Biochar is a carbon-rich and environmentally stable material produced through the pyrolysis of biomass under anaerobic conditions (Khan et al., 2020). Its physical and chemical properties depend on type of feedstock and the pyrolysis conditions, which influence its adsorption capacity, water retention ability, and nutrient content. In recent years, biochar has attracted attention for its dual role in seed coating, serving both as an active protective component and as a solid carrier material. A patented biochar-based seed coating process involving tumbling seeds in a starch or binder solution, followed the addition of biochar to create a uniform layer (Głodowska et al., 2016). Although biochar pelleting can be more expensive than conventional seed pelleting due to higher production costs (Meyer et al., 2011), it support sustainable agriculture by repurposing agricultural waste and contributing to a circular economy. Overall, biochar-based seed coatings represent an innovative strategy for enhancing seed performance, reducing herbicide sensitivity, and promoting environmental sustainability.

Biochar-based seed coatings reduce herbicide injury through several well-documented mechanisms. The porous structure and high surface area of biochar allow for strong adsorption of herbicide molecules, creating a localized zone of reduced herbicide availability around the seed (Zhelezova et al., 2017; Sun et al., 2025). This adsorption slows the movement of active ingredients toward the germinating embryo, thereby lowering phytotoxic exposure. Biochar provides a buffering effect by moderating fluctuations in herbicide concentration and improving micro-environmental conditions for seedling emergence (Khorram et al., 2016; Zhang et al., 2022). The coating itself forms a physical barrier that delays herbicide infiltration, effectively separating the seed from herbicide-treated soil during early germination. Together, these adsorption, buffering, and barrier effects explain why biochar-coated seeds exhibit reduced herbicide stress across systems, including soybean (Sil et al., 2025).

Research studies suggest that seed coatings containing biochar or activated carbon can improve herbicide tolerance and assist in management of invasive weeds (Madsen et al., 2014; Davies et al., 2017; Davies, 2018). Madsen et al. (2014) pioneered the concept of ‘herbicide protection pods’, which combine activated-carbon banding with the precision of seed coatings to shield target plants from herbicide uptake while leaving weeds unprotected. For example, Davies (2018) found that embedding native perennial grass species seeds into biochar-based extruded pellets resulted in higher seedling densities under imazapic application, whereas exotic annual weeds were suppressed. Likewise, grass species commonly used in restoration coated with biochar successfully germinated in a lab setting treated with a pre-emergent herbicide that effectively controlled invasive annual grasses (Holfus et al., 2021). More recently, Terry et al. (2021) compared biochar coating and furrow applications combined with imazapic treatment and found that the dual strategy significantly reduced herbicide damage to seedling emergence, plant density, and growth. Although many of these studies originate from rangeland and ecological restoration systems, their relevance to soybean cropping lies in the shared biological principle: biochar or activated carbon creates a localized herbicide-adsorption zone around the seed, reducing crop phytotoxicity while retaining herbicidal activity against surrounding weeds. This mechanism is independent of species or ecosystem type, making such findings relevant to soybean grown under pre-emergence herbicide regimes.

Although biochar has been extensively investigated for its roles in soil amelioration and herbicide adsorption, its potential application in enhancing herbicide tolerance in soybean through seed coating remains largely underexplored. To date, no field-based study has systematically assessed whether biochar-coated soybean seeds can mitigate pre-emergence herbicide stress while simultaneously improving crop performance and weed suppression. Most existing studies have focused on grasses and rangeland species, leaving a significant research gap in soybean, a crop that is highly sensitive to herbicide-induced phytotoxicity. This gap is particularly critical given the increasing reliance on herbicides in soybean-based cropping systems and the severe yield penalties associated with early-season weed competition. To address this knowledge gap, the present study investigated the performance of biochar-coated soybean seeds under different pre-emergence herbicide regimes. Specifically, the study aimed to (i) evaluate the effects of biochar seed coating on weed dynamics, crop vigor, and yield attributes; (ii) examine the interactive influence between biochar coatings and herbicide applications; and (iii) identify optimal biochar-herbicide combinations for sustainable and efficient soybean production. It was hypothesized that biochar-coated soybean seeds, when combined with pre-emergence herbicides—particularly s-metolachlor + pendimethalin—would alleviate herbicide-induced phytotoxicity, enhance weed suppression efficiency, and improve yield and seed quality compared with uncoated seeds. The outcomes of this study are expected to provide novel insights into the use of biochar-based seed coating as an eco-friendly and sustainable technology for improving herbicide tolerance and overall productivity in soybean cultivation.

## 2 Materials and methods

### 2.1 Study area description

The experiment was conducted during the 2022 and 2023, growing seasons at the Agronomic Research Farm, Department of Agronomy, University of Agriculture, Faisalabad, Pakistan (31.4504° N, 73.1350° E; altitude 186.4 m). The site experiences a semi-arid subtropical climate. Total annual rainfall was 380 mm in 2022 and 345 mm in 2023, with minimum temperatures of 11.2°C and 13.0°C, and maximum temperatures of 25.0°C and 27.0°C, respectively. The experimental soil was classified as sandy clay loam, with a pH of 7.7 in 2022 and 7.5 in 2023. The soil contained 0.13% and 0.16% total nitrogen, and 0.89% and 0.95% organic matter in 2022 and 2023, respectively.

### 2.2 Experimental design and treatments

The experiment was conducted using a randomized complete block design (RCBD) with a factorial arrangement and three replications, comprising a total of 12 treatments. Two seed types were evaluated: biochar-coated and uncoated (control) seeds. These were tested under six weed management strategies: s-metolachlor + pendimethalin, s-metolachlor alone, fluazifop-p-butyl, haloxyfop-p-ethyl, a weed-free treatment, and a weedy check. In the weed-free treatment, weeds were manually removed at weekly intervals throughout the growing period to maintain a weed-free environment.

### 2.3 Biochar preparation and characterization

Biochar was prepared from maize pith residues obtained locally and pyrolyzed under limited oxygen conditions at ~500°C, following standard protocols for agricultural biochar production. The resulting biochar was ground to fine powder (< 200 μm) and subsequently incorporated into the soybean seed-coating protocol with gum arabic as an adhesive agent to ensure uniform adherence on the seed surface. For each 100 g of soybean seed, 15 g of fine biochar powder was applied (15% w/w loading rate), mixed with 5 mL of a 10% gum arabic solution to form a consistent coating layer. Seed coating was performed using a rotary seed coater (Model: JMD-120, Jiangsu Jiuma) operated at 35–40 rpm to ensure uniform material distribution. After coating, seeds were dried in a forced-air drying chamber at 30–35°C for 24 hours and then stored in airtight aluminum-laminated pouches at 20–22°C and 35–40% relative humidity until sowing.

To ensure reproducibility, the physicochemical properties of maize pith biochar were characterized prior to its use. The biochar contained 55–75% carbon, 2–5% hydrogen, 15–30% oxygen, and 0.5–2.5% nitrogen. Ash content ranged from 5–15%, while fixed carbon was 60–75% and volatile matter 10–25%. It exhibited a pH of 7–10, surface area of 50–250 m² g^-^¹, and cation exchange capacity of 50–150 cmol kg^-^¹, consistent with literature values for maize-derived biochar. The moisture content was 2–8%. The ash fraction was enriched with essential minerals, including potassium (1–3%), calcium (0.5–2%), magnesium (0.3–1%), silicon (1–4%), and phosphorus (0.1–0.5%). These properties indicate high sorption potential, alkaline buffering, and mineral nutrient contribution, which are relevant to its role as a protective seed coating against herbicide-induced stress.

### 2.4 Sowing methods

Soybean variety AARI was sown manually in rows spaced 30 cm apart with an intra-row spacing of 7 cm at a seeding rate of 100 kg ha^-^¹. Each experimental plot measured 6 m × 2.1 m, and the net harvested area for yield determination was 5 m × 1.5 m, obtained by excluding the outer border rows and 0.5 m from each end of the plot to minimize edge effects. Only the central rows were used for grain yield measurements, while border rows were excluded from all yield-related analyses to ensure accuracy and reduce border interference.

### 2.5 Data collection

Soybean growth was assessed through destructive sampling at 30 DAS and at 15 days intervals thereafter. A one-foot-square area from each plot was harvested and converted to a one-meter-square equivalent. Samples were separated into leaves and stems, weighed fresh, and subsamples (10 g) were used to determine leaf area using a LI-3100C meter. The remaining plant material was sun-dried for two days and then oven-dried at 65 ± 2°C until a constant weight was achieved. From these data, total dry matter (TDM), leaf area index (LAI), leaf area duration (LAD), crop growth rate (CGR), and net assimilation rate (NAR) were calculated using standard methods. In addition, weed density, weed dry weight, seed yield, and quality traits (protein and oil content) were recorded.

### 2.6 Growth parameter measurement

Crop vigor was visually evaluated at 105 days after sowing (DAS) using a 0–10 rating scale, where 0 denoted plots with dead or least vigorous crops and 10 represented the most vigorous and healthy crop stands. This assessment was conducted following the procedure described by Nikoa et al. (2015). The crop growth rate (CGR) was determined using the standard formula to quantify the rate of dry matter accumulation over the growth period.


$$CGR=\frac{W_{2\;}-\;W_{1}}{t2-t1}$$

Here, W_1_ and W_2_ represent the dry weight measurements at two successive sampling times, T_1_ (initial measurement at 30 DAS) and T_2_ (subsequent intervals at every 15 days).

The leaf area index (LAI), an important indicator of canopy development and plant growth efficiency, was determined using the following formula


$$\text{LAI }=\;\frac{leaf\;\;area}{land\;\;area}$$

Leaf area duration (LAD) was calculated based on the recorded data. Where LAI_1_ and LAI_2_ represent the leaf area indices measured at the respective time intervals t1 and t2.


$$LAD=\frac{\left(LAI1+LAI2\right)\left(t2-t1\right)}{2}$$

To determine leaf area per plant, ten randomly selected tagged plants from each treatment were measured. The length (L) and width (W) of the terminal leaflet were recorded, and their product was adjusted using a leaf shape correction factor to accurately estimate the actual leaf area. This data was then used to calculate the leaf area index (LAI), a crucial parameter for evaluating soybean growth and canopy development. Additionally, the net assimilation rate (NAR) was calculated to assess the rate of dry matter accumulation per unit of leaf area, representing the net increase in plant biomass after accounting for respiratory losses. The NAR was computed using the standard formula.


$$NAR=\frac{TDM}{LAD}$$

### 2.7 Weed assessment

The weed cover score was visually assessed before weed removal using a 1–10 scale, where 1 indicated a completely weed-free condition and 10 represented complete weed coverage, following the method described by Adigun et al. (2017). Weed density was determined by counting all weed species within a 1 m2 quadrat randomly placed at three locations in each plot. Measurements were recorded at four intervals: before herbicide application (initial density), and at 15, 30 and days after spray (DAS). Weeds within the sampled quadrates were cut at ground level, oven-dried at 70°C for 72 hours, and their dry weight was recorded in grams per square meter (g m-2). Weed indices, including weed control efficiency and other standard procedures as follows: The weed control efficiency (WCE), were also calculated. The WCE was expressed as a percentage reduction in weed dry weight in treated plots relative to the control (weedy check) and was computed using the formula:


$$\text{WCE}=\frac{{\text{W}}_{C}-{\text{W}}_{\text{T}}}{\;\;\;{\text{W}}_{\text{C}}}\times \;100$$

The weed index (WI) was calculated using two values—the yield from the weed free plot (YWF) and the yield from the treated plot (YT). The WI was determined using the following formula:


$$\text{WI}=\frac{{\text{Y}}_{\text{WF}}-{\text{Y}}_{\text{T}}}{\;\;\;{\text{Y}}_{\text{WF}}}\times 100$$

The Weed Persistence Index (WPI) was calculated using four parameters: the weed dry weight in the control (unweeded) plot (WC), the weed dry weight in the treated plot (WT), the weed population in the control plot (WPC), and the weed population in the treated plot (WPT). The WPI was computed using the following formula:


$$\text{WPI}=\frac{{\text{W}}_{\text{T}}}{{\text{W}}_{\text{C}}}\times \frac{{\text{W}}_{\text{PC}}}{{\text{W}}_{\text{pT}}}$$

The weed management index (WMI) was calculated using four key parameters: the yield of the treated plot (YT), the yield of the control (unweeded) plot (YC), the weed dry weight in the control plot (WC), and the weed dry weight in the treated plot (WT):


$$\text{WMI}=\frac{\frac{{\text{Y}}_{{\text{T}}^{-\;}}{\text{Y}}_{\text{C}\;}}{{\text{Y}}_{\text{C}\;}}}{\frac{{\text{W}}_{\text{C}}-{\text{W}}_{\text{T}}}{{\text{W}}_{\text{C}}}}$$

The agronomic management index (AMI) was calculated using four parameters: the yield of the treated plot (YT), the yield of the control (unweeded) plot (YC), the weed dry weight in the control plot (WC), and the weed dry weight in the treated plot (WT):


$$\text{AMI}=\frac{\frac{{\text{Y}}_{\text{T}}-{\text{Y}}_{\text{C}}}{{\text{Y}}_{\text{c}}}-\frac{{\text{W}}_{\text{C}}-{\text{W}}_{\text{T}}}{{\text{W}}_{\text{C}}}}{\frac{{\text{W}}_{\text{C}}-{\text{W}}_{\text{T}}}{{\text{W}}_{\text{C}}}}$$

### 2.8 Statistical analysis

All recorded data were analyzed using Statistix 8.1. As the two experimental years exhibited differences in weather conditions and herbicide response trends, the data for each year were analyzed separately, rather than using a combined ANOVA. This approach ensured clearer interpretation of treatment effects without confounding year-to-year environmental variability. Therefore, Year was not included as a factor, and no Year-related interactions (Seed Type × Herbicide × Year) were evaluated. For each year, data were subjected to Fisher’s Analysis of Variance (ANOVA) under a randomized complete block design. When treatment effects were significant, means were separated using the Least Significant Difference (LSD) test at the 5% probability level (P ≤ 0.05). Correlation analyses were performed to evaluate associations among seed-coating treatments, herbicide effects, and growth or yield attributes. Graphical outputs were generated using Origin 2024b.

## 3 Results

### 3.1 Weed flora and infestation patterns

The composition and intensity of weed infestation varied among species and between years (**Supplementary Table S1**). In 2022, *Trianthema portulacastrum* and *Convolvulus arvensis* exhibit the highest infestation levels (60-90%), whereas *Parthenium hysterophorus* showed low infestation (1-29%). In 2023, *Tribulus terrestris* and *Xanthium strumarium* were moderately infested (30-59%), while *Euphorbia hirta* and *Parthenium hysterophorus* maintained low infestation levels. Among grass weeds, *Cynodon dactylon* and *Phalaris minor* showed high infestation (60-90%), in both years. The sedge *Cyperus rotundus* also exhibited persistently high infestation (60-90%), whereas *Cyperus esculentus* declined from moderate (30-59%) in 2022 to low (1-29%) in 2023. *Cyperus difformis* was absent in 2022 but appeared with low infestation (1-29%) in 2023.

### 3.2 Impact of herbicide treatments on leaf area and biomass accumulation

**Figure 1:**
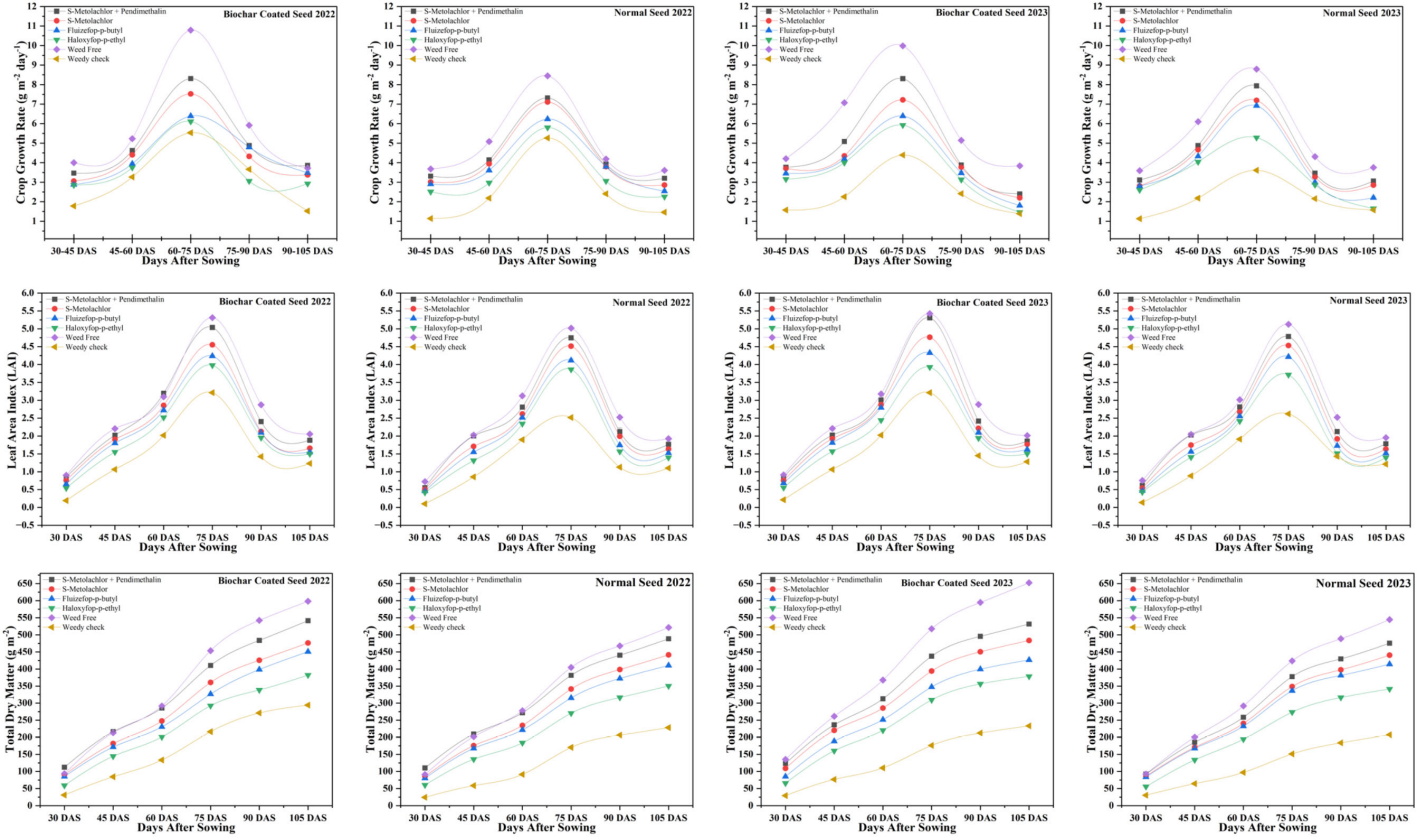
Effect of herbicide treatments on leaf area index (LAI), total dry matter (TDM) and crop growth rate (CGR) in biochar-coated and normal soybean seeds during 2022 and 2023.

Biochar-coated seeds treated with s-metolachlor + pendimethalin consistently exhibited the highest LAI at 75 DAS in both years (5.04 in 2022 and 5.31 in 2023), whereas the lowest values occurred under haloxyfop-p-ethyl at 105 DAS (1.50 and 1.51 in 2022 and 2023, respectively). In 2023, LAI of biochar-coated seeds under s-metolachlor + pendimethalin was 10.9% higher than that of normal seeds under the same treatment, while even under the least favorable conditions (haloxyfop-p-ethyl, 105 DAS), LAI remained 23.9% higher than normal seeds in the weedy check (**Figure 1**). Total dry matter (TDM) accumulation followed a similar trend, with biochar-coated seeds achieving the maximum under s-metolachlor + pendimethalin at 105 DAS (541.49 g m^-^² in 2022 and 531.60 g m^-^² in 2023) and the minimum under haloxyfop-p-ethyl at 30 DAS (59.07 g m^-^² and 65.87 g m^-^², respectively). Relative to normal seeds, biochar-coated seeds accumulated 10.7% more TDM under optimal conditions and up to 11.1% more even under the least favorable treatment (**Figure 1**).

The maximum CGR for biochar-coated seeds occurred under s-metolachlor + pendimethalin during the 60–75 DAS (8.31 g m^-2^ day^-1^), while the lowest was recorded under haloxyfop-p-ethyl during 75–90 DAS (2.85 g m^-2^ day^-1^). Comparable trends were observed for normal seeds, though with generally lower values. Across herbicide treatment and growth intervals, biochar coating improved maximum CGR by 2.7–16.0% over normal seeds (**Figure 1**). Biochar-coated seeds under s-metolachlor + pendimethalin also recorded the highest crop vigor (6.70–7.13 in 2022; 7.03–7.13 in 2023), cumulative leaf area duration (189.7–210.4 in 2022; 195.6–211.3 in 2023), and net assimilation rate (2.47–2.57 in 2022; 2.47–2.52 in 2023). In contrast, the lowest values were consistently observed in normal seeds treated with haloxyfop-p-ethyl. Weed-free plots outperformed weedy checks across all parameters and treatments, showing 36–67% higher crop vigor, 56–78% greater leaf area duration, and 31–56% higher net assimilation rates (**Supplementary Table S2**).

### 3.3 Weed density, suppression efficiency, and control indices

**Figure 2:**
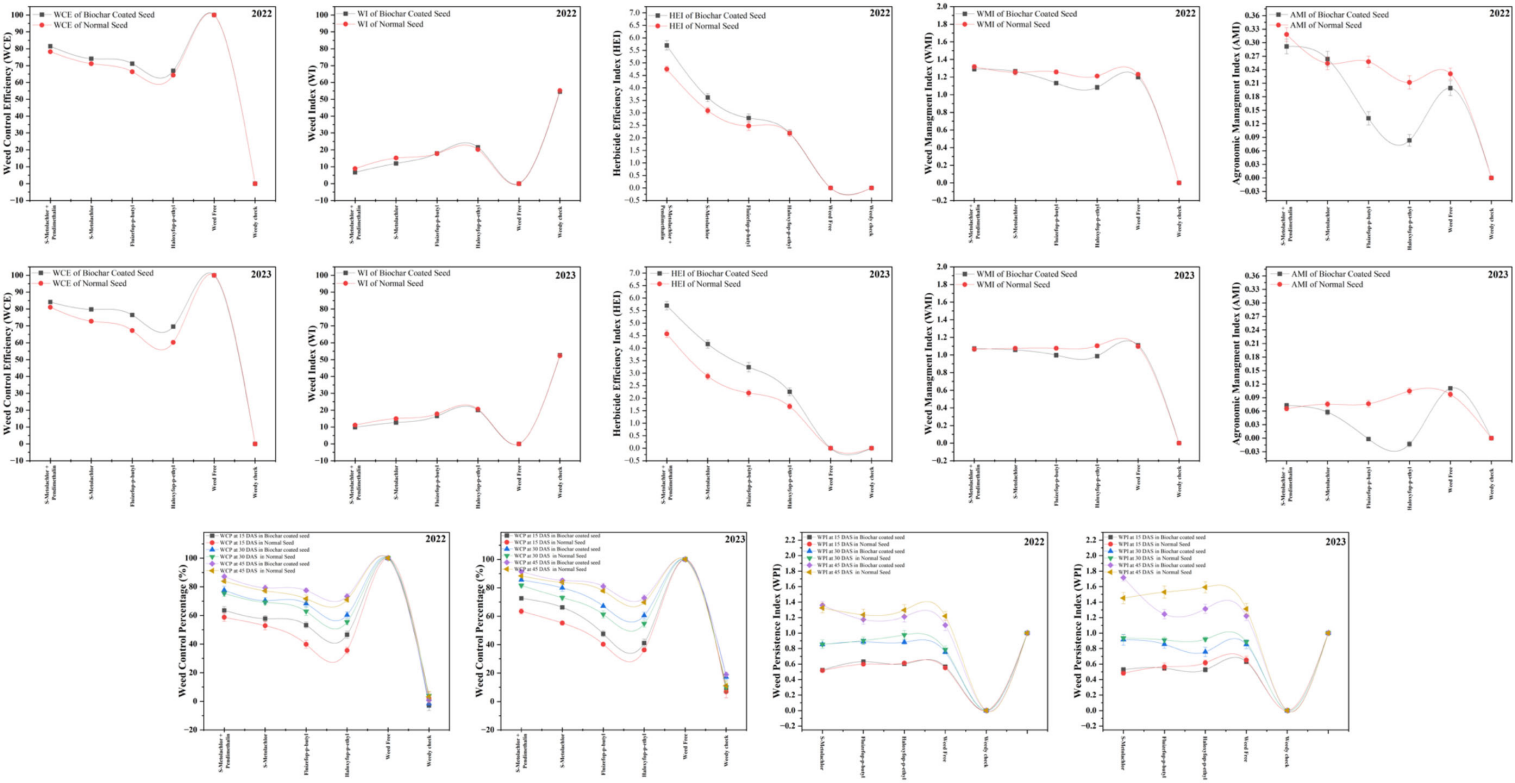
Comparison of weed control efficiency (WCE), weed index (WI), herbicide efficiency index (HEI), weed management index (WMI), agronomic management index (AMI), weed control percentage (WCP) and weed persistence index (WPI) of different herbicides in biochar-coated and normal soybean seeds in 2022 and 2023.

Pre-emergence application of s-metolachlor + pendimethalin to biochar-coated soybean seeds proved most effective for weed suppression across all growth stages (**Supplementary Table S3**). At 15 DAS, weed density in these plots ranged from 104.3 to 129.0 weeds m^-2^ in 2022 and 55.3 to 104.3 weeds m^-2^ in 2023. Densities declined progressively, reaching 55.3–79.3 weeds m^-^² at 30 DAS and 29.7–48.7 weeds m^-^² at 45 DAS in both years. Across herbicide treatments, biochar-coated seeds consistently recorded 12.5% lower weed densities than normal seeds, with reductions of 19.2%, 15.6%, and 14.8% at 15, 30, and 45 DAS, respectively. The WCP peaked under s-metolachlor + pendimethalin at 45 DAS, attaining 87.1% in 2022 and 91.4% in 2023, while the lowest values occurred under haloxyfop-p-ethyl at 15 DAS (46.5% 41.1%, respectively) (**Figure 2**).

On average, biochar-coated seeds achieved 3.1–8.3% higher WCP than normal seeds. Similarly, the weed performance index (WPI) reached maximum values of 1.36 (2022) and 1.72 (2023) under s-metolachlor + pendimethalin, whereas the lowest values were recorded under s-metolachlor at 30 DAS. Biochar coating increased WPI by about 3% over normal seeds. The weed control efficiency (WCE) followed a comparable trend, highest under s-metolachlor + pendimethalin (81.5% in 2022 and 84.1% in 2023). The lowest WCE values occurred under haloxyfop-p-ethyl. Averaged across treatments, biochar-coated seeds exhibited a 3.3% improvement in WCE. Correspondingly, the weed index (WI) remained consistently lower in biochar-coated seeds (6.7–21.6%) than in normal seeds (8.9–20.2%), with the lowest WI observed under s-metolachlor + pendimethalin (**Figure 2**).

The herbicidal efficacy index (HEI) also peaked with s-metolachlor + pendimethalin for biochar-coated seeds (5.7% in both years), exceeding normal seeds by about 1%. The weed management index (WMI) was likewise highest in this treatment (1.29 in 2022 and 1.07 in 2023) and lowest under haloxyfop-p-ethyl. Although biochar-coated seeds showed a 2.3% decrease in WMI compared to normal seeds, the agronomic management index (AMI) exhibited a 10.5% improvement, with maximum values of 0.2917 (2022) and 0.0725 (2023) under s-metolachlor + pendimethalin. Assessment of susceptibility index, weed cover score, and weed dry weight at harvest (**Table 1**) further confirmed the superior weed suppression achieved by biochar-coated seeds treated with s-metolachlor + pendimethalin. This treatment recorded the lowest susceptibility indices (1.53 in 2022 and 1.20 in 2023), minimal weed cover scores (2.4 and 2.1), and the least weed dry weights (699.49 kg ha^-^¹ and 516.16 kg ha^-^¹). In contrast, haloxyfop-p-ethyl on normal seeds resulted in the highest susceptibility indices (8.50 and 7.5) and weed dry weights (1412.15 and 1214.54 kg ha^-^¹). Under weed-free conditions, complete weed suppression was achieved (susceptibility index, weed cover, and weed dry weight all 0.00), confirming total herbicidal efficacy. Conversely, under weedy check conditions, biochar-coated seeds exhibited a 29.7% higher susceptibility index and a 558.3% increase in weed dry weight compared with the most effective herbicide treatment. Normal seeds under weedy check showed even greater values, with 5.7% higher susceptibility, 8.6% higher weed cover, and 60.3% greater weed biomass than biochar-coated seeds. Overall, these results highlight that biochar-coated seeds combined with s-metolachlor + pendimethalin consistently outperformed all other treatments, ensuring superior weed suppression, reduced biomass accumulation, and improved crop-weed balance across both seasons (**Figure 2**).

**Table 1:** Impact of herbicide treatments on susceptibility index, weed cover, and weed dry weight in biochar-coated and normal soybean seeds during 2022 and 2023.

Treatments		Time of application	Dose (ml acre^-1^)	Susceptibility index		Weed cover score		Weeds dry weight at harvest	
				2022	2023	2022	2023	2022	2023
Seed treatment	Herbicides								
Biochar Coated Seed	S-Metolachlor + Pendimethalin	PRE	900 ml acre^-1^	1.53 ± 0.09^h^	1.2 ± 0.12^f^	2.4 ± 0.09^g^	2.1 ± 0.09^h^	699.49 ± 12.92^j^	516.16 ± 14.68^i^
	S-Metolachlor	PRE	800 ml acre^-1^	2.50 ± 0.06^g^	2.2 ± 0.06^f^	3.4 ± 0.06^f^	3.1 ± 0.06^g^	980.32 ± 10.12^h^	659.54 ± 13.54^g^
	Fluizefop-p-butyl	POST	800 ml acre^-1^	3.27 ± 0.09^f^	3.1 ± 0.09^e^	4.2 ± 0.06^e^	3.8 ± 0.06^f^	1092.06 ± 13.51^g^	766.95 ± 16.45^f^
	Haloxyfop-p-ethyl	POST	350 ml acre^-1^	4.33 ± 0.09^d^	3.8 ± 0.06^d^	5.2 ± 0.06^d^	4.1 ± 0.06^e^	1251.19 ± 13.19^e^	990.81 ± 13.57^d^
	Weed Free	–	–	0.00 ± 0.00^i^	0.00 ± 0.00^h^	0.00 ± 0.00^h^	0.00 ± 0.00^i^	0.00 ± 0.00^k^	0.00 ± 0.00^j^
	Weedy check	–	–	0.00 ± 0.00^i^	0.00 ± 0.00^h^	8.5 ± 0.09^b^	7.8 ± 0.09^a^	3784.83 ± 8.84^b^	3254.85 ± 12.59^a^
Normal Seed	S-Metolachlor + Pendimethalin	PRE	900 ml acre^-1^	3.80 ± 0.06^e^	3.3 ± 0.06^e^	3.7 ± 0.09^f^	3.2 ± 0.06^g^	860.00 ± 10.67^i^	577.59 ± 13.41^h^
	S-Metolachlor	PRE	800 ml acre^-1^	4.70 ± 0.06^c^	4.2 ± 0.03^c^	4.5 ± 0.09^e^	3.7 ± 0.06^f^	1141.61 ± 10.66^f^	830.67 ± 14.48^e^
	Fluizefop-p-butyl	POST	800 ml acre^-1^	6.37 ± 0.09^b^	5.5 ± 0.09^b^	5.1 ± 0.06^d^	4.7 ± 0.03^d^	1332.67 ± 13.99^d^	999.96 ± 10.10^d^
	Haloxyfop-p-ethyl	POST	350 ml acre^-1^	8.50 ± 0.06^a^	7.5 ± 0.12^a^	5.8 ± 0.06^h^	5.3 ± 0.09^c^	1412.15 ± 8.96^c^	1214.54 ± 11.72^c^
	Weed Free	–	–	0.00 ± 0.00^i^	0.00 ± 0.00^h^	0.00 ± 0.00^h^	0.00 ± 0.00^i^	0.00 ± 0.00^k^	0.00 ± 0.00^j^
	Weedy check	–	–	0.00 ± 0.00^i^	0.00 ± 0.00^h^	9.0 ± 0.12^a^	7.2 ± 0.06^b^	3958.13 ± 12.38^a^	3051.60 ± 13.95^b^
	MS for seed treatment (S)			34.4178**	26.0100**	4.1344**	2.5600**	201008**	59063**
	MS for herbicides (H)			40.0840**	31.4684**	49.6647**	36.5213**	10,320,000**	7079754**
	MS for S × H			4.2011**	3.1227**	0.2778**	0.7033**	9482.83**	41804**
	LSD At 5%			0.1635	0.1942	0.3874	0.1793	26.927	25.902

Means followed by different superscript lowercase letters within a column are significantly different at p ≤ 0.05 according to LSD test. ** indicates a statistically significant effect at p ≤ 0.01.

### 3.4 Soybean growth and phenological traits

**Figure 3:**
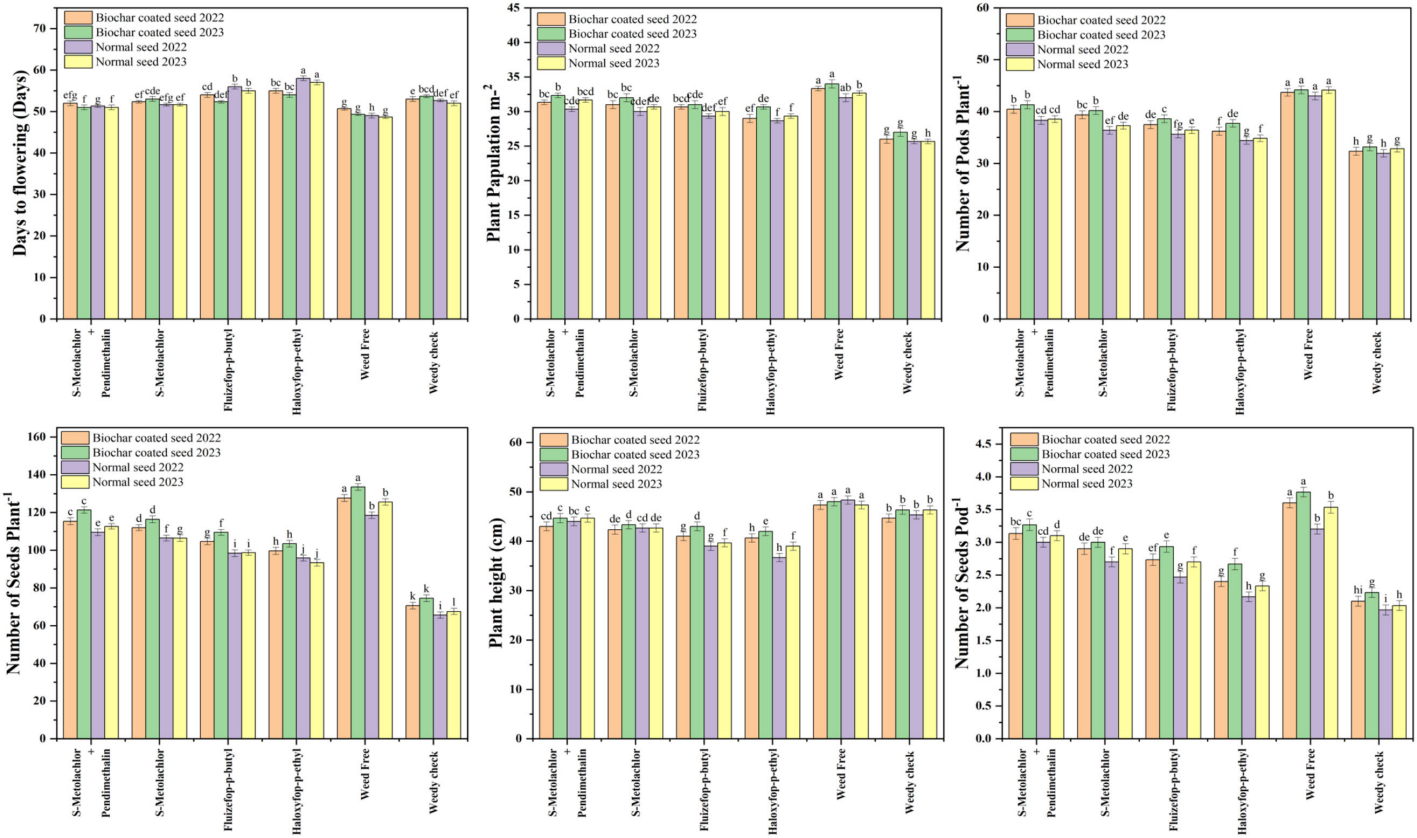
Impact of herbicide treatments on soybean growth and yield parameters in biochar-coated and normal seeds during 2022 and 2023.

In 2022, the pre-emergence (PRE) application of s-metolachlor + pendimethalin produced the highest plant population, reaching 31.33 plants m^-2^ for biochar-coated seeds and 30.33 plants m^-2^ for normal seeds. The lowest population was observed under haloxyfop-p-ethyl (POST), with 29.00 plants m^-2^ and 28.67 plants m^-2^, respectively. Biochar coating increased the plant population by 3.29% under the s-metolachlor + pendimethalin treatment. A similar trend was observed in 2023, where the same treatment again resulted in the maximum plant stand (32.33 vs. 31.67 plants m^-^² for biochar-coated and normal seeds, respectively) (**Figure 3**). Days to flowering varied among treatments and years. In 2022, the longest duration was observed for biochar-coated seeds under haloxyfop-p-ethyl (55 days), while in 2023, the maximum was recorded with fluazifop-p-butyl (52.33 days). The shortest flowering time for biochar-coated seeds occurred under s-metolachlor + pendimethalin (52 and 51 days in 2022 and 2023). For normal seeds, flowering was earliest under weed-free conditions (49 and 48.67 days). Plant height followed a similar pattern, with the tallest plants observed under s-metolachlor + pendimethalin PRE for biochar-coated seeds (43.00 cm in 2022 and 44.67 cm in 2023), while the shortest were recorded under haloxyfop-p-ethyl POST for normal seeds (36.67 cm and 39.00 cm, respectively). Biochar-coated seeds under s-metolachlor + pendimethalin showed a 2.48% decrease in plant height relative to normal seeds in 2022, but a 5.63% increase in 2023. Under haloxyfop-p-ethyl, biochar-coated seeds exhibited a 10.18% higher plant height in 2022, which shifted to a 7.69% decrease in 2023 (**Figure 3**). These contrasting trends between years likely reflect differences in early-season environmental conditions, which are known to influence both herbicide persistence and biochar–herbicide interactions. Higher soil moisture and temperature in one year may enhance herbicide mobility, allowing biochar to adsorb more active ingredient and reduce crop injury, whereas drier or cooler conditions in the other year may limit biochar’s buffering capacity, leading to reduced plant height.

### 3.5 Yield components and seed productivity

**Figure 4:**
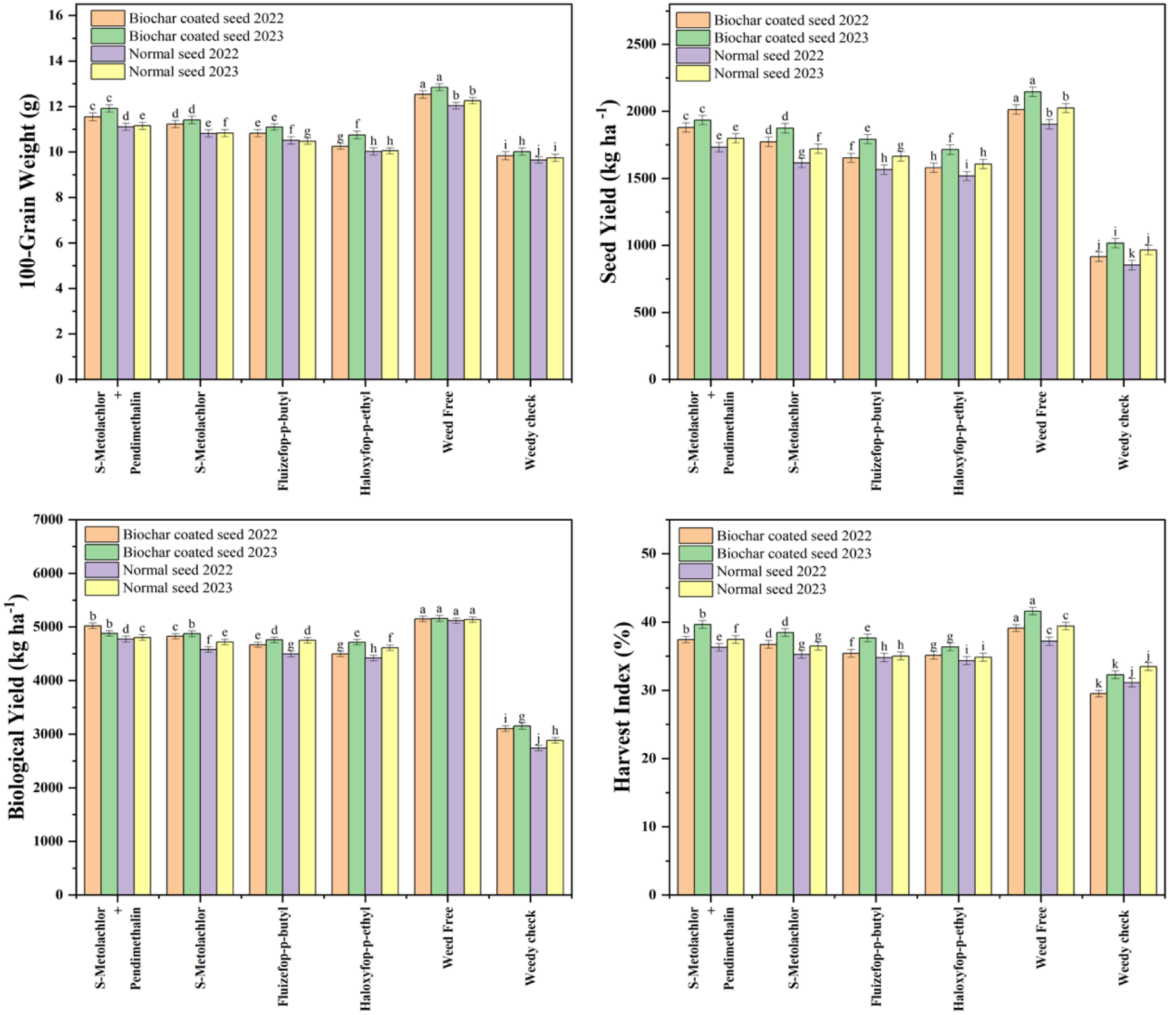
Impact of herbicide treatments on soybean 100-grain weight, seed yield, biological yield and harvest index in biochar-coated and normal seeds during 2022 and 2023.

In both years, the pre-emergence application of s-metolachlor + pendimethalin to biochar-coated seeds produced the highest yield and yield components. This treatments resulted in the maximum number of pods per plant (40.43 and 41.30), seeds per pod (3.13 and 3.27), seeds per plant (115.33 and 121.33), 100-grain weight (11.54 and 11.91 g), seed yield (1879.21 and 1933.77 kg ha^-1^), stover yield (3141.65 and 2944.92 kg ha^-1^), biological yield (5020.87 and 4878.69 kg ha^-1^), and harvest index (37.43% and 39.64%), in 2022 and 2023, respectively. Conversely, the post-emergence (POST) application of haloxyfop-p-ethyl to normal seeds resulted in the lowest values for these parameters in both years. Biochar coating generally improved yield attributes under most herbicide regimes. Under s-metolachlor + pendimethalin PRE, biochar-coated seeds produced 6–7% more pods per plant, 4% more seeds per pod, and 4–6% higher 100-grain weight than normal seeds. However, under weed-free conditions, the number of pods per plant slightly declined (≈7%) for biochar-coated seeds. Compared with the weedy check, seed yield under s-metolachlor + pendimethalin PRE increased by about 51% in 2022 and 48% in 2023, while the weed-free plots showed similar gains (45–48%) over the weedy check. Stover yield showed minor year-to-year variation, with biochar-coated seeds under s-metolachlor + pendimethalin PRE performing slightly better in 2022 (+3.4%) but marginally lower in 2023 (−1.3%) compared to normal seeds. Fluazifop-p-butyl POST improved stover yield by 3–4% for biochar-coated seeds across both years. Comparing the best (s-metolachlor + pendimethalin PRE) and worst (haloxyfop-p-ethyl POST) treatments, biological yield for biochar-coated seeds increased by 11.7% in 2022 and 3.5% in 2023, while normal seeds showed 8% and 4% increases, respectively. The harvest index followed a similar pattern, improving by 3–6% under the most effective treatment relative to the least effective one (**Figures 3**, **4**).

### 3.6 Seed quality attributes (protein, oil content, and oil yield)

Biochar-coated seeds showed higher protein content, oil content, and oil yield than normal seeds in both years. Pre-emergence (PRE) application of s-metolachlor + pendimethalin produced the highest quality parameters, with protein reaching 35.72%, oil content 19.84%, and oil yield 381.75 kg ha^-^¹. While post-emergence (POST) application of haloxyfop-p-ethyl resulted in the lowest values for all seed quality traits. Weed-free plots also outperformed the weedy check, with 15–25% greater protein, 17–21% higher oil content, and 35–38% higher oil yield. Across both years, biochar-coated seeds showed a 2.3% increase in protein and 1.5% increase in oil content, contributing to an average 54.2 kg ha^-^¹ improvement in oil yield compared with normal seeds (**Table 2**).

**Table 2:** Soybean quality response to various herbicides application in biochar coated and normal seed during 2022 and 2023.

Treatments		Time of application	Dose (ml acre^-1^)	Protein content (%)		Oil content (%)		Oil yield (kg ha^-1^)	
				2022	2023	2022	2023	2022	2023
Seed treatment	Herbicides								
Biochar Coated Seed	S-Metolachlor + Pendimethalin	PRE	900 ml acre^-1^	34.70 ± 0.15^c^	35.29 ± 0.10^d^	19.35 ± 0.05^c^	19.84 ± 0.08^c^	361.58 ± 0.99^c^	381.75 ± 1.14^c^
	S-Metolachlor	PRE	800 ml acre^-1^	33.70 ± 0.09^e^	34.87 ± 0.06^e^	18.80 ± 0.08^d^	19.57 ± 0.09^c^	331.18 ± 1.85^d^	364.70 ± 2.82^d^
	Fluizefop-p-butyl	POST	800 ml acre^-1^	33.16 ± 0.10^f^	33.59 ± 0.10^g^	18.69 ± 0.08^d^	18.77 ± 0.10^d^	307.01 ± 1.67^f^	334.29 ± 1.66^e^
	Haloxyfop-p-ethyl	POST	350 ml acre^-1^	32.41 ± 0.12^g^	33.10 ± 0.07^h^	18.32 ± 0.06^e^	18.46 ± 0.08^e^	287.24 ± 1.46^g^	314.44 ± 1.28^f^
	Weed Free	–	–	36.32 ± 0.08^a^	37.16 ± 0.09^a^	20.62 ± 0.10^a^	20.96 ± 0.09^a^	413.10 ± 0.98^a^	447.69 ± 0.81^a^
	Weedy check	–	–	30.77 ± 0.11^j^	31.71 ± 0.09^j^	16.56 ± 0.09^i^	16.79 ± 0.12^i^	149.62 ± 1.60^j^	168.62 ± 1.15^j^
Normal Seed	S-Metolachlor + Pendimethalin	PRE	900 ml acre^-1^	34.12 ± 0.09^d^	35.72 ± 0.12^c^	18.30 ± 0.11^e^	18.66 ± 0.12^de^	315.10 ± 1.85^e^	333.64 ± 2.64^e^
	S-Metolachlor	PRE	800 ml acre^-1^	32.81 ± 0.10^f^	34.29 ± 0.08^f^	17.87 ± 0.08^f^	17.94 ± 0.07^f^	286.39 ± 1.31^g^	306.57 ± 0.57^g^
	Fluizefop-p-butyl	POST	800 ml acre^-1^	31.70 ± 0.10^h^	33.17 ± 0.06^h^	17.58 ± 0.08^g^	17.62 ± 0.13^g^	273.11 ± 2.18^h^	290.98 ± 1.21^h^
	Haloxyfop-p-ethyl	POST	350 ml acre^-1^	31.17 ± 0.10^i^	32.62 ± 0.09^i^	17.14 ± 0.09^h^	17.28 ± 0.14^h^	258.19 ± 1.27^i^	275.70 ± 2.02^i^
	Weed Free	–	–	35.58 ± 0.22^b^	36.26 ± 0.08^b^	19.83 ± 0.08^b^	20.28 ± 0.09^b^	375.35 ± 2.59^b^	408.41 ± 1.78^b^
	Weedy check	–	–	29.68 ± 0.13^k^	30.57 ± 0.09^k^	16.15 ± 0.09^j^	16.44 ± 0.11^j^	135.74 ± 1.55^k^	156.61 ± 1.55^k^
	MS for seed treatment (S)			9.0451**	2.3881**	7.45472**	9.5357**	10593.9**	14349.8**
	MS for herbicides (H)			24.2698**	23.7895**	9.73685**	11.0061	42658.3**	46382.0**
	MS for S × H			0.1568**	0.4327**	0.11734**	0.3012**	214.5**	357.2**
	LSD At 5%			0.3517	0.2320	0.2539	0.3051	4.9781	4.5554

Means followed by different superscript lowercase letters within a column are significantly different at p ≤ 0.05 according to LSD test. ** indicates a statistically significant effect at p ≤ 0.01.

### 3.7 Correlation analysis effects between seed coating and herbicide treatments

**Figure 5:**
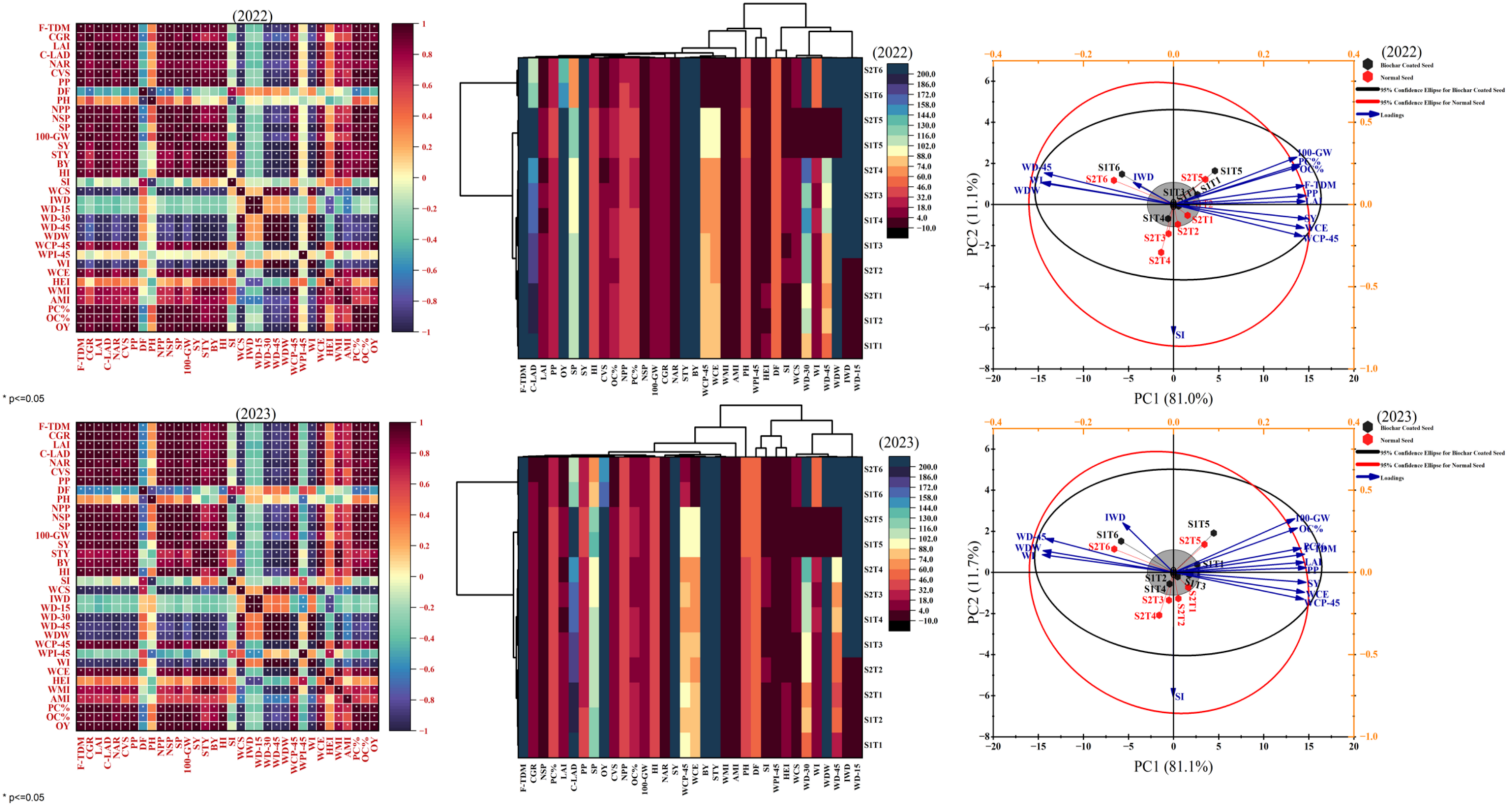
Pearson correlation, clustered heatmap, principal component analysis (PCA) of weeds attributes and soybean growth, yield, and quality parameters treated with biochar coated and normal seed under different herbicides application during 2022 and 2023. F-TDM, final total dry matter; CGR, crop growth rate; LAI, leaf area index; LAD, leaf area duration; NAR, net assimilation rate; CVS, crop vigor score; PP, plant population; DF, days to flowering; PH, plant height; NPP, number of pods per plant; NSP, number of seeds per pod; SP, number of seeds per plant; 100-GW, 100 grains weight; SY, seed yield; STY, stover yield; BY, biological yield; HI, harvest index; SI, susceptibility index; WCS, weed cover score; IWD, initial weed density; WD-15, weeds density after 15 days of spray; WD-30, weeds density after 30 days of spray; WD-45, weeds density after 45 days of spray; WDW, weeds dry weight; WCP-45, weed control percentage at 45-das; WPI-45, weed persistence index at 45 -das; WI, weed index; WCE, weed control efficiency; HEI, herbicide efficiency index; WMI, weed management index; AMI, agronomic management index; PC, protein content; OC, oil content; OY, oil yield.

Pearson correlation analyses for 2022 and 2023 revealed consistent relationships among weed attributes, soybean growth, yield, and quality parameters. Yield components such as SY, BY, and 100-grain weight exhibited positive correlations (warmer color gradients) with growth parameters including CGR, LAI, and NAR, indicating that vigorous vegetative growth translated into higher productivity. In contrast, weed densities (WD-15, WD-30, WD-45) showed strong negative correlations (cooler colors) with yield and growth traits, confirming the suppressive impact of increased weed competition on soybean performance. Weed control efficiency (WCE) was significantly and positively associated with yield attributes, highlighting the importance of effective weed suppression. Likewise, protein and oil contents correlated positively with yield, suggesting that improved growth not only enhanced productivity but also seed quality. Clustered heatmaps provided additional insight into treatment similarities. In 2022, biochar-coated seeds (S1) and normal seeds (S2) treated with s-metolachlor + pendimethalin (PRE) and haloxyfop-p-ethyl (POST) (S1T6 and S2T6) formed closely related clusters because of comparable responses in final total dry matter (F-TDM), LAI, and protein content (PC%). Treatments involving fluazifop-p-butyl (S1T3, S1T5) showed greater variability, particularly in WCE and initial weed densities. A similar clustering trend appeared in 2023, where S1T6 and S2T6 again grouped together, reflecting analogous influences on seed yield and oil yield, whereas treatments like S2T3 and S2T4 diverged markedly due to higher susceptibility indices and weed cover scores. Principal component analysis (PCA) corroborated these patterns. The first principal component (PC1) accounted for the majority of total variance (81.0% in 2022 and 81.1% in 2023), dominated by strong positive loadings of F-TDM, CGR, LAI, and SY—parameters closely linked to vigorous crop growth and efficient weed management. Biochar-coated seeds consistently outperformed normal seeds across these components. Conversely, weed-related variables including WD-45 and weed dry weight showed negative loading on PC1, linking them with reduced crop performance. Treatments T1 (s-metolachlor + pendimethalin, PRE) and T5 (weed-free) aligned with superior growth and yield vectors, while T6 (weedy check) was positioned in the opposite quadrant, characterized by higher weed pressure and reduced performance (**Figure 5**).

## 4 Discussion

### 4.1 Biochar-seed coating reduces herbicide injury

Weed infestation remains a major constraint to soybean production, as weeds compete with the crop for nutrients, water, and light, resulting in substantial yield losses. The present study revealed considerable variation in weed infestation among species, underscoring the importance of effective and integrated weed management strategies. Broadleaf weeds such as *Trianthema portulacastrum*, *Convolvulus arvensis*, *Tribulus terrestris*, *Xanthium strumarium*, *Euphorbia granulata*, *Euphorbia hirta*, and *Parthenium hysterophorus* exhibited diverse infestation patterns, reflecting their ecological adaptability to changing environments and management practices. Among them, *Trianthema portulacastrum* and *Convolvulus arvensis* showed particularly high infestation levels, posing serious challenges to soybean productivity. In contrast, *Parthenium hysterophorus* exhibited relatively low infestation, likely due to its greater sensitivity to environmental factors and targeted control measures (Menalled et al., 2001).

Among grass weeds, *Cynodon dactylon* and *Phalaris minor* emerged as the most persistent species, maintaining consistently high infestation levels in both years. Their strong competitive ability and rapid regrowth potential make them particularly problematic for soybean cultivation. These results emphasize the necessity of integrated approaches capable of managing both broadleaf and grass weeds simultaneously. Similarly, sedge species, such as *Cyperus rotundus*, *Cyperus esculentus*, and *Cyperus difformis*, contributed significantly to the overall weed flora (Daramola et al., 2019). *Cyperus rotundus*, known for its rhizomatous growth and resilience, maintained high infestation levels across seasons, demonstrating its resistance to conventional control methods. In contrast, *Cyperus esculentus* and *Cyperus difformis* exhibited more variable infestation patterns, suggesting greater responsiveness to environmental fluctuations and herbicide management (Daramola, 2020).

Biochar seed coating has been reported to improve germination and seedling establishment of desirable species even under challenging conditions (Williams et al., 2015). However, few practical and cost-effective methods exist for enhancing crop establishment in heavily weed-infested fields, where weeds rapidly colonize and suppress crop emergence (Stanbury et al., 2018). Effective weed control during early growth stages is therefore critical to prevent reduced crop survival, altered vegetation composition, and nutrient imbalances (Fisher et al., 2006; Singh et al., 2014a, b). Recent advances in seed coating technologies—particularly those incorporating biochar or activated carbon—offer promising solutions. When applied prior to herbicide use, these coatings can shield seeds and emerging seedlings from herbicide injury, improving establishment and early growth (Madsen et al., 2014; Davies et al., 2017; Davies, 2018; Arshad et al., 2025c). These protective effects of biochar coatings not only mitigate herbicide injury but also create a foundation for enhanced soybean growth and yield, as further discussed in the next section.

### 4.2 Enhancement of soybean growth and yield

Biochar seed coatings effectively mitigated the adverse effects of herbicides on key growth parameters, including seedling emergence, crop vigor score, TDM, LAI, CGR, LAD, and NAR (Terry et al., 2021). Among these parameters, LAI served as a sensitive indicator of soybean canopy development and physiological responses to herbicide stress and weed competition (Camara-Williams, 2019). Pre-emergence herbicides, such as s-metolachlor + pendimethalin, promoted early weed control, allowing soybean plants to allocate more resources to canopy development and photosynthesis (Holfus et al., 2021). Consequently, higher LAI values were recorded during critical growth stages, particularly flowering and pod filling, when efficient light interception is essential for maximizing photosynthesis and yield potential. In contrast, post-emergence herbicides such as haloxyfop-p-ethyl induced physiological stress, suppressing leaf expansion and canopy development, thereby reducing LAI, light interception, and overall productivity.

Clenet et al. (2019) reported similar findings while exploring restoration strategies for weed-invaded communities. By integrating native seeds into activated carbon pods alongside indaziflam herbicide, they demonstrated that high-performance pellets (HPPs) protected seeded species from herbicide injury, enhancing plant abundance and size compared to unprotected seeds. This integrated strategy demonstrated a promising method for restoring annual communities invaded by weeds. Biochar seed coatings can influence not only LAI but broader crop growth performance. Biochar enhances soil fertility, water retention, and nutrient availability, thereby promoting root development and improving nutrient uptake in soybean plants (Camara-Williams, 2019). As a result, soybean grown from biochar-coated seeds showed improved canopy development and higher LAI compared to normal seeds. The variation in LAI across growth stages and herbicide treatments further underscores biochar’s potential in enhancing crop resilience to environmental stress (Głodowska et al., 2017), although its effectiveness may vary with soil properties, climatic conditions, and management practices.

Similarly, TDM data reflected cumulative biomass accumulation under different herbicide regimes. Pre-emergence herbicides facilitated early weed suppression, enabling greater resource allocation to aboveground biomass production and resulting in higher TDM during periods of active vegetative growth and canopy expansion (Holfus et al., 2021). Conversely, delayed weed control under post-emergence herbicides, prolonged competition and reduced biomass accumulation in soybean plants (Rupareliya et al., 2020). This resulted in lower TDM and overall productivity due to persistent weed interference and herbicide stress. Holfus et al. (2021) also observed that biochar-coated seeds substantially outperformed normal seeds under herbicide application, with a 307% increase in emergence, a 235% increase in seedling height, and an 87% increase in biomass. CGR data further supported these findings, as pre-emergence herbicides promoting effective early weed control enhanced biomass accumulation and canopy expansion rates (Ezebuiro et al., 2021a). In contrast, post-emergence herbicides reduced CGR by delaying canopy development and impairing physiological processes (Madsen et al., 2014). These differences in CGR among herbicide treatments and seed types illustrate the interactive effects of weed control efficacy, herbicide stress, and plant physiological adaptation.

Overall, biochar-coated seeds exhibited higher crop vigor scores, greater cumulative LAD, and enhanced NAR compared to normal seeds, confirming their benefits in promoting seedling establishment and sustained early plant growth (Chen, 2021). These results are consistent with earlier findings demonstrating biochar’s role in improving soil fertility, moisture retention, and nutrient use efficiency (Camara-Williams, 2019; Rupareliya et al., 2020; Arshad et al., 2025c). The observed enhancements in crop vigor, LAD, and NAR underscore the potential of biochar-based seed-coating as a practical strategy to enhance crop performance and resilience under both weed competition and herbicide-induced stress (Głodowska et al., 2017). Since growth and yield responses are closely linked to weed dynamics, it is therefore necessary to further examine how biochar seed coatings influence weed control indices and efficiency.

### 4.3 Improved weed control indices and efficiency

The effects of herbicide treatments on weed density and control efficacy revealed significant variations between biochar-coated and normal seeds. Pre-emergence herbicides, particularly s-metolachlor + pendimethalin, provided superior weed suppression, reducing overall weed density and improving crop performance compared to post-emergence herbicides, such as haloxyfop-p-ethyl (Holfus et al., 2021). These findings align with previous research highlighting that herbicide timing and application method are critical determinants of weed control efficiency and yield protection (Aher et al., 2023; Arshad et al., 2025c). Key weed indices, including susceptibility index, weed cover score, and weed dry weight showed significant responses to both herbicide type and seed coating. Among the evaluated treatments, s-metolachlor + pendimethalin exhibited the highest efficacy, reflected in lower susceptibility indices and weed cover scores (Ezebuiro et al., 2021b). These findings reinforce the importance of selecting broad-spectrum, low-phytotoxicity herbicides tailored to weed species composition, growth stage, and environmental conditions. Pre-emergence herbicides also promoted higher plant populations and earlier flowering, supporting improved crop establishment and reproductive growth, consistent with earlier findings on herbicide impacts on phenology and yield components (Głodowska et al., 2017; Daramola, 2020). Baughman et al. (2021) introduced the herbicide protection pod (HPP) technology, showing that smaller HPPs doubled emergence rates and improved biomass compared to larger pellets while maintaining herbicide protection. This suggests that tailored HPP size can optimize outcomes across various environments. Comparisons of plant attributes and yield parameters between biochar-coated and normal seeds under different herbicides demonstrated the benefits of seed coating for enhancing productivity (Głodowska et al., 2017). Biochar-coated seeds with s-metolachlor + pendimethalin consistently achieved higher plant height, pod number, seeds per pod, 100-grain weight, seed yield, stover yield, biological yield, and harvest index compared to normal seeds, especially under post-emergence herbicides. Comparable observations were reported by Holfus et al. (2021), who noted that biochar-coated seeds improved emergence by 307%, seedling height by 235%, and biomass by 87% under herbicide exposure. These results confirm that integrating biochar seed coating with PRE herbicides enhances early growth, resource utilization, and overall yield potential (Camara-Williams, 2019).

Biochar-coated seeds likely improved crop performance by increasing soil water retention, nutrient availability and microbial activity, thereby promoting root development, nutrient uptake, and stress tolerance (Terry et al., 2021; Chen, 2021). These mechanisms explain the superior yield attributes observed in biochar-coated seeds with s-metolachlor + pendimethalin (Ezebuiro et al., 2021b; Holfus et al., 2021; Arshad et al., 2025b). In addition, biochar mitigates herbicide phytotoxicity by adsorbing excessive herbicide residues, thereby reducing injury (Kadam et al., 2018). Pre-emergence herbicides provide early weed suppression, which enhances resource availability, root establishment, and biomass accumulation (Rupareliya et al., 2020). Consequently, biochar-coated seeds combined with pre-emergence herbicides exhibited higher yields and better growth traits compared to those treated with post-emergence herbicides, In contrast, post-emergence herbicides may adversely affect photosynthesis, nutrient assimilation, and reproductive processes, ultimately reducing yield and quality (Madsen et al., 2014; Kadam et al., 2018). Conversely, post-emergence herbicides were comparatively less effective, resulting in greater yield losses from prolonged weed competition (Clenet et al., 2019; Rupareliya et al., 2020; Arshad et al., 2025a). Madsen et al. (2014) similarly demonstrated that activated carbon or biochar coating enhanced emergence by 4.8-fold, height by 3.8-fold, and biomass by 19-fold under higher herbicide stress.

Indices such as weed control efficiency, herbicide efficiency index (HEI), and weed management index (WMI) further confirmed the advantages of biochar-coated seeds treated with s-metolachlor + pendimethalin, which exhibited superior weed suppression and agronomic performance compared to normal seeds. These finding suggests biochar improves herbicide retention, soil moisture, and root growth, enhancing persistence and weed suppression (Williams et al., 2015). Similarly, Brown et al. (2019) reported that biochar pelleting reduced seedling mortality from 96% to 22% under pre-emergent herbicides, indicating its protective role in promoting seedling establishment and ecosystem restoration. Overall, elevated HEI, WMI, and agronomic management index (AMI) values observed in biochar-coated seeds highlight improved resource efficiency, cost-effectiveness, and environmental sustainability. These results affirm that integrating biochar seed coating with pre-emergence herbicides represents a promising and sustainable approach for optimizing soybean productivity and weed management (Smith et al., 2016; Baughman et al., 2021; Arshad et al., 2025d).

### 4.4 Effects on seed quality and environmental implications

Beyond growth and yield, biochar-coated seeds consistently exhibited higher protein content, oil content, and oil yield across all herbicide treatments than normal seeds, highlighting their potential to improve the nutritional quality of soybean grains. These improvements can be attributed to enhanced soil fertility, nutrient cycling, and nutrient uptake, which promote protein synthesis and lipid accumulation in soybean grains. The combination of biochar seed coating with pre-emergence herbicides, therefore, offers a practical strategy to improve not only productivity but also grain nutritional quality and economic value in soybean-based systems. In addition, biochar seed coating reduced direct herbicide contact with emerging seedlings and improved yield stability by enhancing soil health and crop resilience to environmental stressors such as drought, temperature fluctuations, and land degradation. However, variations in AMI suggest that interactions among herbicide type, biochar properties, and environmental conditions can influence treatment effectiveness. Future studies should therefore evaluate the long-term effects of biochar seed coatings on soil microbial diversity, nutrient cycling, and ecosystem services. Biochar-coated seeds may also contribute to ecological restoration by facilitating crop and native plant establishment while suppressing invasive weeds, particularly when integrated with suitable agronomic practices. Nevertheless, broader testing across different soil types, climates, and crop species is required before large-scale adoption, as some species may remain sensitive to pre-emergence herbicides without optimized protection (Kyser et al., 2013).

Overall, this study underscores the pivotal role of integrated weed management in enhancing soybean productivity and quality. Among the tested treatments, biochar-coated seeds combined with s-metolachlor + pendimethalin provided the most effective weed suppression, highest yield, and superior grain quality. This approach represents a climate-smart, environmentally sound, and economically viable strategy for improving soybean resilience under weed pressure. Future efforts should focus on optimizing biochar particle size, coating methods, and application rates, while integrating these innovations with precision herbicide use, conservation tillage, and cover cropping. Addressing socioeconomic barriers to adoption will further support the development of sustainable and resilient soybean production systems.

## 5 Conclusions

This study demonstrated that biochar-coated soybean seeds performed better than uncoated seeds across multiple agronomic and quality parameters under pre- and post-emergence herbicide applications. Biochar coating reduced herbicide-induced injury, improved early crop vigor, and enhanced plant growth traits, including plant height, pod formation, and overall seed yield. The combination of biochar-coated seeds with the pre-emergence herbicide s-metolachlor + pendimethalin consistently produced the most favorable results by improving weed suppression, reducing susceptibility to herbicide stress, and achieving higher grain protein, oil content, and oil yield. Although the weed-free treatment produced the highest yield, biochar-coated seeds under s-metolachlor + pendimethalin closely matched its performance while outperforming the weedy check in both years. These findings confirm that biochar seed coating can mitigate crop injury from herbicides and enhance soybean productivity under typical field conditions. Further research should refine coating formulations and evaluate performance across different environments and soybean varieties.

## Data Availability

The original contributions presented in the study are included in the article/**Supplementary Material**. Further inquiries can be directed to the corresponding authors.
