# Supplementary material for: Enhancing soybean tolerance to pre-emergent herbicides via biochar seed coating for eco-safe food systems
Source: Front Plant Sci. 2026 Jan 19;16:1700864. doi: 10.3389/fpls.2025.1700864 (PMC12862483; doi:10.3389/fpls.2025.1700864)
Supplement: Supplementary file 1 [file Table1.docx]

Supplementary Material

# Supplementary Tables

**
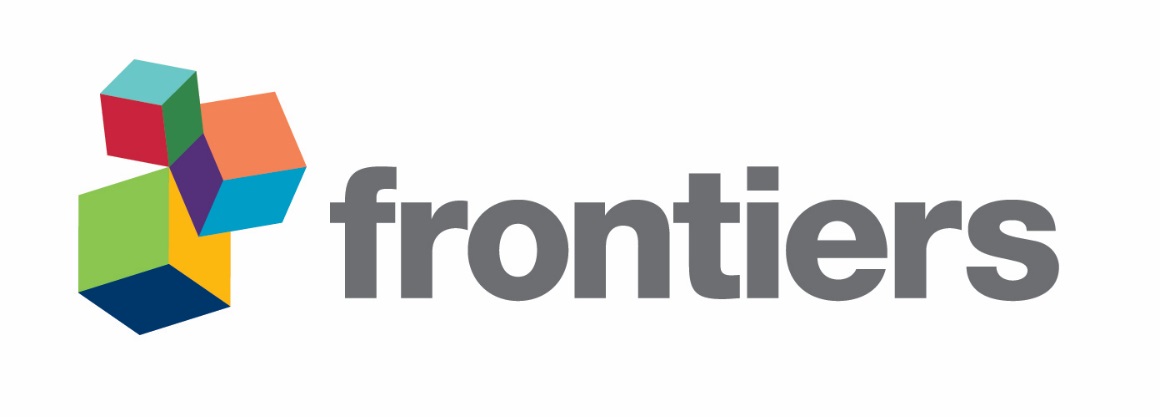
**

**Supplementary Tables**

**Table S1. Weed species and their level of infestation during the period of crop growth in 2022 and 2023.**

| **Weed species** | **Plant family** | **Level of infestation** | |
| --- | --- | --- | --- |
|  |  | **2022** | **2023** |
| **Broad leaf weeds** | | | |
| Trianthema portulacastrum (Linn). | Aizoaceae | *** | *** |
| *Convolvulus arvensis* (Linn). | [*Convolvulaceae*](http://www.misin.msu.edu/facts/?project=misin&kdetail=Plants&duration=NA&habit=NA&fdetail=Morning%20glory) | *** | *** |
| *Tribulus terrestris* L. | *Zygophyllaceae* | *** | ** |
| *Xanthium strumarium* L. | *Compositae* | ** | ** |
| *Euphorbia granulate* L. | *Euphorbiaceae* | ** | * |
| *Euphorbia hirta* L. | *Euphorbiaceae* | - | * |
| *Parthenium hysterophorus* L. | *Asteraceae* | * | * |
| **Grasses** | | | |
| *Cynodon dactylon* (L) Gaertn | *Poaceae* | *** | *** |
| *Paspalum distichum* (L). | *Poaceae* | - | * |
| Phalaris minor Retz. | Poaceae | * | * |
| **Sedges** | | | |
| *Cyperus rotundus* | *Cyperaceae* | *** | *** |
| *Cyperus esculentus* | *Cyperaceae* | * | - |
| *Cyperus difformis* | *Cyperaceae* | - | * |

******* Highly infested (60-90); ****** Moderately infest (30-59%); * Low infestation (1-29%); **^-^** not noticeable.

Table S2. Effects of various herbicide applications on soybean growth parameters for biochar-coated and normal seeds during 2022 and 2023.

| **Treatments** | | **Time of application** | **Dose** | **Crop Vigor Score** | | **Cumulative Leaf Area Duration (LAD)** | | **Net Assimilation Rate (NAR)** | |
| --- | --- | --- | --- | --- | --- | --- | --- | --- | --- |
|  |  |  |  | **2022** | **2023** | **2022** | **2023** | **2022** | **2023** |
| **Seed Treatment** | **Herbicides** |  |  |  | | | | | |
| Biochar Coated Seed | S-Metolachlor + Pendimethalin | PRE | 900 ml acre^-1^ | 6.70±0.06^c^ | 7.13±0.09^c^ | 210.35±0.30^b^ | 211.34±0.44^b^ | 2.57±0.00^b^ | 2.52±0.01^c^ |
|  | S-Metolachlor | PRE | 800 ml acre^-1^ | 6.20±0.12^c^ | 7.03±0.09^d^ | 189.72±0.46^d^ | 195.64±0.24^c^ | 2.51±0.00c^d^ | 2.47±0.01^d^ |
|  | Fluizefop-p-butyl | POST | 800 ml acre^-1^ | 5.77±0.09^d^ | 6.70±0.12^e^ | 179.39±0.74^e^ | 181.65±0.65^e^ | 2.51±0.01^cd^ | 2.35±0.01^e^ |
|  | Haloxyfop-p-ethyl | POST | 350 ml acre^-1^ | 5.23±0.09^e^ | 6.30±0.06^f^ | 165.37±0.22^f^ | 163.55±0.02^h^ | 2.31±0.01^f^ | 2.31±0.02^e^ |
|  | Weed Free | - | - | 7.70±0.12^a^ | 8.20±0.06^a^ | 224.50±0.45^a^ | 227.69±0.54^a^ | 2.66±0.01^a^ | 2.86±0.00^a^ |
|  | Weedy check | - | - | 3.20±0.06^i^ | 3.63±0.09^i^ | 126.52±0.77^h^ | 127.43±0.43^j^ | 2.33±0.02^f^ | 1.83±0.01^g^ |
| Normal Seed | S-Metolachlor + Pendimethalin | PRE | 900 ml acre^-1^ | 5.70±0.12^d^ | 6.70±0.06^e^ | 192.57±1.89^c^ | 194.18±0.13^d^ | 2.54±0.03^c^ | 2.45±0.00^d^ |
|  | S-Metolachlor | PRE | 800 ml acre^-1^ | 5.10±0.06^f^ | 5.90±0.06^f^ | 178.58±0.33^e^ | 179.42±0.62^f^ | 2.47±0.01^e^ | 2.45±0.01^d^ |
|  | Fluizefop-p-butyl | POST | 800 ml acre^-1^ | 4.57±0.03^g^ | 4.77±0.09^g^ | 163.75±0.53^f^ | 165.83±0.52^g^ | 2.51±0.02^cd^ | 2.50±0.02^c^ |
|  | Haloxyfop-p-ethyl | POST | 350 ml acre^-1^ | 3.73±0.09^h^ | 4.33±0.09^h^ | 149.89±0.99^g^ | 149.45±0.20^i^ | 2.34±0.01^f^ | 2.28±0.00^f^ |
|  | Weed Free | - | - | 7.30±0.06^b^ | 7.70±0.12^b^ | 210.21±0.14^b^ | 210.93±0.52^b^ | 2.48±0.02^de^ | 2.58±0.01^b^ |
|  | Weedy check | - | - | 2.23±0.09^j^ | 2.40±0.12^j^ | 105.06±0.15^i^ | 112.47±0.74^k^ | 2.18±0.01^g^ | 1.84±0.02^g^ |
|  | **MS for seed treatment (S)** |  |  | 9.5069** | 12.9600** | 2293.13** | 2257.68** | 0.03868** | 0.01361** |
|  | **MS for herbicides (H)** |  |  | 15.7556** | 17.0867** | 7675.39** | 7499.68** | 0.10357** | 0.53424** |
|  | **MS for S × H** |  |  | 0.1969** | 0.6627** | 18.01** | 1.96* | 0.01065** | 0.03117** |
|  | **LSD At 5%** |  |  | 0.2636 | 0.2526 | 2.1972 | 1.4247 | 0.0363 | 0.0239 |

**Table S3. Effect of Herbicide Treatments on Weed Density in Biochar-Coated and Normal Soybean Seeds at Various Intervals in 2022 and 2023.**

| **Treatments** | | **Time of application** | **Dose (ml acre-1)** | **Initial Weed density Before Spray** | | **Weed density at 15 days after spray** | | **Weed density at 30 days after spray** | | **Weed density at 45 days after spray** | |
| --- | --- | --- | --- | --- | --- | --- | --- | --- | --- | --- | --- |
|  |  |  |  | **2022** | **2023** | **2022** | **2023** | **2022** | **2023** | **2022** | **2023** |
| **Seed Treatment** | **Herbicides** |  |  |  | | | | | | | |
| Biochar Coated Seed | S-Metolachlor + Pendimethalin | PRE | 900 ml acre^-1^ | 0.00±0.00^f^ | 0.00±0.00^d^ | 129.00±5.69^f^ | 104.33±3.48^g^ | 79.33±6.69^f^ | 55.33±4.63^f^ | 48.67±4.33^e^ | 29.67±4.10^g^ |
|  | S-Metolachlor | PRE | 800 ml acre^-1^ | 0.00±0.00^f^ | 0.00±0.00^d^ | 149.33±5.61^e^ | 129.00±6.43^f^ | 105.00±3.79^de^ | 76.00±6.35^e^ | 78.00±5.51^d^ | 50.67±3.84^ef^ |
|  | Fluizefop-p-butyl | POST | 800 ml acre^-1^ | 373.33±10.17^bcd^ | 299.00±10.44^c^ | 174.67±8.09^d^ | 156.33±6.17^de^ | 118.33±6.49^cd^ | 98.33±4.33^d^ | 84.00±4.04^cd^ | 56.33±4.33^de^ |
|  | Haloxyfop-p-ethyl | POST | 350 ml acre^-1^ | 398.00±11.27^b^ | 286.00±7.23^c^ | 212.33±4.48^c^ | 168.00±4.04^de^ | 157.67±4.91^b^ | 112.67±3.48^c^ | 106.00±5.51^b^ | 77.33±4.06^c^ |
|  | Weed Free | - | - | 347.67±11.85^de^ | 364.00±10.39^ab^ | 0.00±0.00^g^ | 0.00±0.00^h^ | 0.00±0.00^g^ | 0.00±0.00^g^ | 0.00±0.00^f^ | 0.00±0.00^h^ |
|  | Weedy check | - | - | 354.00±9.81^cde^ | 381.67±13.38^a^ | 363.67±3.76^a^ | 347.67±6.89^a^ | 359.33±6.17^a^ | 315.33±4.33^a^ | 351.00±4.16^a^ | 309.00±4.62^a^ |
| Normal Seed | S-Metolachlor + Pendimethalin | PRE | 900 ml acre^-1^ | 0.00±0.00f^e^ | 0.00±0.00^d^ | 155.00±6.56^e^ | 125.33±3.48^f^ | 92.67±4.91^ef^ | 63.33±4.91^f^ | 60.67±5.49^e^ | 40.33±3.18^fg^ |
|  | S-Metolachlor | PRE | 800 ml acre^-1^ | 0.00±0.00^f^ | 0.00±0.00^d^ | 177.33±6.17^d^ | 154.00±4.04^e^ | 116.33±3.76^cd^ | 92.67±5.55^d^ | 85.67±4.91^cd^ | 55.33±4.63^de^ |
|  | Fluizefop-p-butyl | POST | 800 ml acre^-1^ | 339.33±13.38^e^ | 285.67±13.30^c^ | 203.00±8.08^c^ | 170.33±4.91^d^ | 125.67±6.64^c^ | 110.00±3.21^c^ | 95.67±6.17^bc^ | 63.33±3.76^d^ |
|  | Haloxyfop-p-ethyl | POST | 350 ml acre^-1^ | 369.67±9.26^cd^ | 307.33±6.64^c^ | 238.00±4.04^b^ | 196.00±8.08^c^ | 164.67±4.98^b^ | 138.67±4.98^b^ | 107.00±4.93^b^ | 92.67±3.18^b^ |
|  | Weed Free | - | - | 443.00±10.44^a^ | 342.33±12.14^b^ | 0.00±0.00^g^ | 0.00±0.00^h^ | 0.00±0.00^g^ | 0.00±0.00^g^ | 0.00±0.00^f^ | 0.00±0.00^h^ |
|  | Weedy check | - | - | 377.00±11.27^bc^ | 344.00±11.30^b^ | 367.67±5.93^a^ | 319.67±6.94^b^ | 362.33±5.55^a^ | 308.67±4.10^a^ | 364.33±4.33^a^ | 305.00±3.79^a^ |
|  | **MS for seed treatment (S)** |  |  | 784^ns^ | 659^ns^ | 3136.0** | 900.0** | 441.0* | 774.7** | 521.4* | 283.4** |
|  | **MS for herbicides (H)** |  |  | 226416** | 175209** | 84849.3** | 70243.3** | 87498.9** | 67561.2** | 93069.4** | 72621.6** |
|  | **MS for S × H** |  |  | 3316** | 625* | 254.1* | 669.0** | 37.2^ns^ | 204.6** | 51.0^ns^ | 74.0^ns^ |
|  | **LSD At 5%** |  |  | 26.248 | 26.441 | 15.371 | 15.069 | 14.258^ns^ | 11.093 | 13.800^ns^ | 10.802^ns^ |
